# Austria

**Published:** 1896-11

**Authors:** 


*Austria* reports the finding of trichina in American pork, but so much chicanery and deception in our foreign meat-products are practised by dealers, even to the extent of removing the canvas coverings from hams and placing the same on home-grown products, that those in charge of the Bureau of Animal Industry are very loath to accept the alleged finding.

